# Explainable models for forecasting the emergence of political instability

**DOI:** 10.1371/journal.pone.0254350

**Published:** 2021-07-29

**Authors:** Emma Baillie, Piers D. L. Howe, Andrew Perfors, Tim Miller, Yoshihisa Kashima, Andreas Beger

**Affiliations:** 1 School of Computing and Information Systems, University of Melbourne, Melbourne, Victoria, Australia; 2 Melbourne School of Psychological Sciences, University of Melbourne, Melbourne, Victoria, Australia; 3 Independent Researcher, Tallinn, Harjumaa, Estonia; Vellore Institute of Technology: VIT University, INDIA

## Abstract

Building on previous research on the use of macroeconomic factors for conflict prediction and using data on political instability provided by the Political Instability Task Force, this article proposes two minimal forecasting models of political instability optimised to have the greatest possible predictive power for one-year and two-year event horizons, while still making predictions that are fully explainable. Both models employ logistic regression and use just three predictors: *polity code* (a measure of government type), *infant mortality*, and *years of stability* (i.e., years since the last instability event). These models make predictions for 176 countries on a country-year basis and achieve AUPRC’s of 0.108 and 0.115 for the one-year and two-year models respectively. They use public data with ongoing availability so are readily reproducible. They use Monte Carlo simulations to construct confidence intervals for their predictions and are validated by testing their predictions for a set of reference years separate from the set of reference years used to train them. This validation shows that the models are not overfitted but suggests that some of the previous models in the literature may have been. The models developed in this article are able to explain their predictions by showing, for a given prediction, which predictors were the most influential and by using counterfactuals to show how the predictions would have been altered had these predictors taken different values. These models are compared to models created by lasso regression and it is shown that they have at least as good predictive power but that their predictions can be more readily explained. Because policy makers are more likely to be influenced by models whose predictions can explained, the more interpretable a model is the more likely it is to influence policy.

## Introduction

In our interconnected world, leaders and decision-makers have an interest in promoting peace and stability in both their own region and also globally. Coupled with the growing amount of available data with which to model the behaviour and outcomes of countries around the world, there is increasing motivation and opportunity to use this data to provide objective and reliable methods for identifying characteristics that give rise to a higher risk of political instability [1]. With that information we hope to be able to predict where instability is more likely to occur and identify potential measures to reduce this risk.

However, such predictions are unlikely to have much influence on policy makers unless they can be understood. The current trend in the field is to create models with ever increasing predictive power [2]. This has resulted in increasingly complex models, with some of the latest models combining several of the earlier models so as to obtain the maximum possible predictive power [3]. Unfortunately, by their very nature, these complex models are hard to explain and therefore may not be trusted by policy makers [4].

The purpose of our study was to create models to predict political instability that have a high predictive power but whose predictions can be readily explained. We did this by creating models where transparency was built into them from the start. These models were as focused on the transparency of their predictions as they were on the accuracy of their predictions. Following the advice of Rudin [5], we constructed these sparse models using meaningful predictors that were combined in an iterative fashion that was designed to restrict the model to the minimum number of predictors to achieve an acceptable level of accuracy.

Compared to other modelling tasks, the modelling of political instability presents some particular challenges. The onset of instability is, fortunately, a rare event, so our conclusions about how to identify it must be based on a small number of instances, and the range of years in which we have reliable and relevant data is relatively short compared to other forecasting fields (e.g. weather forecasting). The identification of optimal methods for dealing with rare events and other unbalanced class data is a subject of active research [6] and previous studies on instability have used a variety of techniques. In addition, there is still no general agreement on the best factors for predicting instability. From early influential models such as those of Fearon and Laitin [7], Collier and Hoefflery [8], the State Failure Task Force (SFTF) [9] and others, to more recent integrated systems such as ICEWS [10] and ViEWS [11], researchers have used a variety of different predictors, including macroeconomic data on a country-year basis, fine-grained geographic data [12] and natural language data based on speeches and other communications of political leaders [13]. In this initial study, we will be confining ourselves to macro-economic (country-year) data as this data is freely available which ensures that our results can be reproduced by other reseachers. In future projects, we will build on our macroeconomic models to investigate whether incorporating additional sources of data can increase predictive accuracy further while maintaining the transparency of the models.

### Defining instability

There are a number of possible definitions of political instability. Previous research has defined it in various ways including civil war (violent conflict leading to some minimum threshold of deaths), breakdown in the ability of government to maintain order, or political upheaval without a required threshold of violent events or deaths [14–16]. In keeping with prior researchers [9, 15, 17]), we have used data on political instability provided by the Political Instability Task Force (PITF)—previously the SFTF—which maintains a database of political instabilities dating from 1948 to the present day. The bulk of this data was originally coded as part of a well-defined research project, with further updates operating under the oversight of the original researchers. Consequently, the criteria used for defining instability has remained consistent and there has been negligible concept drift. Instabilities are divided into Revolutionary Wars, Genocides and Politicides (mass killings based on either ethnic group membership or political status), Ethnic Wars (ethnic conflict with violence on both sides) and Adverse Regime Change (coups and similar state failures including non-violent transitions to more authoritarian forms of government) [18]. Because there are relatively few instances of each type of instability, our model collapses across these categories to predict the probability that political instability occurs, regardless of its type. We justify this by the fact that increases in the probability of one type of instability typically lead to increases in the probability of others, which shows that these different types of instability share at least some common causes [19]. In addition, had we considered each type of instability individually, there would not have been a sufficient number of instability events to make reliable predictions; when we tried modelling each instability type separately our resultant models had poor performance. While we note that other studies have confined themselves to predicting only one type of instability (e.g. [7, 8]), these studies were likely overfitted given the limited data set, the fact that these studies had eight and ten predictors respectively, and did not have a separate test set.

For our study, we constructed models to predict instability within the period 1976–2017. For this period there were 109 instabilities out of a total of 5421 available country-years. Thus, the base rate for instabilities was just 2.0%. Of these instabilities, 42% involved Adverse Regime Change, 28% involved Revolutionary War, 41% involved Ethnic War and 9% involved GenoPoliticide, including some instabilities of multiple types.

### Previous models

There is a substantial literature on creating predictive models of political instability using country-year data on macro-economic factors. Table 1 briefly summarises this literature, excluding models that either did not use only country-year data, did not use only macroeconomic predictors or did not explicitly specify their predictors (e.g. [2, 3, 20, 21]). From this table it is clear that there is little consensus as to which macroeconomic predictors should be used or even how many predictors should be used, with the number of predictors ranging from three for the SFTF (1995) up to ten for Collier and Hoeffler (2004). With regards the latter point, we note the ‘Rule of Three’ proposed by Achen [22], which recommends that, in a linear or logistic model, three should be the maximum number of predictive factors to allow for an accurate analysis of the actual effects of these factors. In Table 1, the only model to satisfy this rule was the one proposed by the SFTF.

**Table 1:** Models of instability.

Reference	Predictors	Dependent Variable	Data Years	Prediction Years
Fearon & Laitin [7]	Ethnic & Religious fractionalisation,	Civil War	1945–1999	none
	GDP per capita, Population,			
	Previous Instability,			
	Mountainous/Non-Contiguous Territory,			
	Oil exporter, New State,			
	Polity Code			
Collier [8]	Primary commodity exports/GDP,	Civil War	1960–1999	none
	Low secondary schooling,			
	Low per-capita income,			
	Low economic growth,			
	Population, Democracy score,			
	Time since previous conflict,			
	Geographic dispersion,			
	Social fractionalisation,			
	Dominance of one ethnic group			
SFTF [9]	Democracy, Trade Openness,	State Failure (later ‘Political Instability’)	1955–1994	none
	Infant Mortality			
King [29]	Democracy, Trade Openness,	Political Instability	1955–1990	1991–1998
	Infant Mortality,			
	Military Population,			
	Population Density,			
	Legislative Effectiveness			
Goldstone [15]	Polity Code, Infant Mortality,	Political Instability	1955–2003	1995–2005
	Conflict in neighboring States,			
	State-led discrimination			
Ulfelder [16]	Population, GDP per capita,	Mass Killing	1945–2011	cross-validated
	Existing Civil War, Anocracy,			
	Post-Cold-War period			
Kennedy [17]	Polity Code, Infant Mortality,	Political Instability	1955–1994	1995–2012
	Conflict in neighboring States,			
	State-led discrimination			
Goldsmith [14]	Democracy/Autocracy,	Genocide/Politicide	1974–1987	1988–2003
	Conflict in neighboring States,			
	Recent Assassinations,			
	Proximity to Election Year,			
	Ethnic Fractionalisation,			
	Years since Previous Genocide			

In addition to the literature discussed above, there is a literature that has investigated how particular individual factors influence the probability of political instability, focusing on factors such as climate change [23], youth bulge under various conditions [24], oil exports, regime ideology [25], membership of international organisations [26] and so on. Consequently, we used this literature to suggest additional potential predictors.

Even with the exclusions and caveats discussed above, to adequately discuss the details of the remaining literature would take a monograph. Instead, we will confine ourselves to discussing just two commonalities to justify the choices we made in developing our models. First, as indicated in Table 1, many of these previous investigations either did not test their predictions or, if they did, used the same data set for both training and testing. As these models often based their predictions on a large number of factors, it is unclear to what extent these models were overfitted to the data, especially given the rarity of instability events. As such, the future performance of some of these models is questionable. This problem is particularly acute in the field of Political Science because this field has not traditionally emphasised the use of tools to estimate confidence intervals, preferring to concentrate on point value predictions [27]. In this project we will test our models on data that were not used to train the models so as to provide the most robust test possible of our model’s future predictive performance. Additionally, we will utilise Monte Carlo simulations to provide confidence intervals for our predictions, which allows us to validate the stability of our overall metric of interest (the model’s general ability to distinguish between instances of high and low instability-probability, as measured by the area under the precision-recall curve—AUPRC) and additionally check the overall fit of the model by comparing expectations to outcomes in binned groups of country-years. The necessity of this work was demonstrated by Bowlsby et al. [28] who showed that models trained in any one specific time period can vary widely in their performance in subsequent years.

The second point is that a number of these previous models relied on infrequently-updated hand-calculated predictors which demand a high level of expert decision-making. Any predictor whose availability may lag by multiple years cannot in practise be used to predict instability in a real-time context. For example, both [15, 17] used *State-led discrimination* as a predictor, which has not been updated since 2009. In our project we confined ourselves to publicly available predictors that are updated regularly.

### Notable features of the present study

Our objective was to create two models to predict political instability one and two years in advance, respectively. The two models developed in the present study have the following notable features:

1. Simplicity: Both models used just three predictors, thereby minimising the risk of overfitting, and enhancing explainability.
2. Reproducibility: Both models used predictors that are publicly available and are updated on a regular basis. This means that our results can be reproduced and new predictions made in a timely manner.
3. Validation: To demonstrate that the models were not overfitted, we tested them using data that was not used in their construction.
4. Error bars: All model predictions were accompanied with error bars, signifying a 95% confidence interval for the predicted probability. Without error bars, model predictions cannot be meaningfully compared [27].
5. Predictions explained: Because models will only influence policymakers if their predictions can be explained, we demonstrate how all our model predictions can be explained.

While there have been a number of other models of political instability, most of them used a large number of predictors. The exceptions were the model created by the SFTF, which used just three predictors [9], and the models proposed by [15, 17], both of which used four predictors. As all the other models used at least five predictors, we believe that they are likely overfitted because we demonstrate that, using similar predictors to those used by those models, only three predictors are needed. Out of the models listed in Table 1, the SFTF model is the most likely to make robust predictions as it contains the fewest number of predictors. We go beyond the SFTF model by validating our models on data that was not used to train them and by including error bars for all our predictions, thereby allowing us to have an appropriate level of confidence in our model predictions. In addition, we used AUPRC whereas the SFTF model used just AUC (area under the receiver operating characteristic curve), which is a less appropriate metric given the low number of instability events [4].

Turning our attention to the models of Goldstone et al and Kennedy, we note that they both used a predictor, *state-led discrimination*, that is no longer publicly available. As such, neither model can continue to be used to make predictions. Conversely, for our models, we confined our attention to publicly available predictors. Equally importantly, we were able to explain each prediction by showing which predictors were the most influential and by using counterfactuals to show how this prediction would have been altered had the predictors taken different values. This allowed a more nuanced understanding of both the data and our models that had been obtained in previous studies. For example, both Goldstone et al. and Kennedy emphasized the predictive power of *polity code*. While we agree that in some circumstances *polity code* has considerable predictive power, we were able to show that in other circumstances its predictive power is minimal and other predictors such as *infant mortality* are more important. Furthermore, by using AUPRC instead of AUC, we were better able to quantify the performance of our models. Finally, as our models used one fewer predictors than both of these models, our models are simpler and likely more robust. For these reasons, we believe that our models represent a significant improvement over previous models in predicting state instabilities based on macroeconomic (country-year) data.

## The models

As the macroeconomic data is available for each country for each year, we will refer to it as country-year data. There is debate over how models should be constructed using such data [17]. Many researchers have used a case/control methodology because in a dataset with rare events, models are most influenced by the rare events themselves—the cases—and very little by each additional non-case data point. However, because we are using existing publicly available data, there is no data-collection cost in simply using all the available data.

A variety of modelling methods can be used including Random Forest, K-nearest neighbor and other non-parametric models. With very noisy data it is often the case that that the simplicity of logistic regression, which reduces the chance of overfitting, is superior [16]. Consequently, our models used only logistic regression.

In our research, we started with a range of possible predictors chosen to fit the following criteria: they are based on publicly available data, where there exists data for at least 85% of country-years covering the period from 1950–2017 (which includes years used for model training as well as prediction) for the 176 countries in our data set, and they have either been found by previous researchers to be significant, or measure similar concepts as factors that were previously found to be important. These predictors were derived from demographic data, financial data, measures of personal welfare, social measures and measures of conflict. Some predictors found by previous researchers to be useful had to be excluded on the grounds of being infrequently updated, or unlikely to be available in the future (e.g. state-led discrimination or legislative effectiveness). A full description of the potential predictors that we used is given in Table A in S1 Text and the correlations between these predictors are shown in Tables B and C in S1 Text.

Because our models are designed to be predictive, they will be evaluated on their predictive performance, as measured by the area under the precision-recall curve (i.e. AUPRC). This allows for a ‘threshold-agnostic’ approach in which models can be ranked independently of a threshold (i.e. probability cutoff) used to define which countries are predicted to become unstable. A high AUPRC shows that a model is ranking all its predictions well, including those with very low and very high likelihood, whereas defining a single threshold treats all countries above (or below) the threshold alike, whether they are only just above, or significantly above the threshold. AUPRC has gained popularity in recent years compared to other measures such as AUC due to its greater ability to discriminate cases in situations where the events of interest are rare [3].

The objective of the modelling was to discover which factors had the greatest predictive power rather than to provide specific numbers for the size of the effect for each of the chosen predictors. In a real-world situation in which decision-makers use a model as a basis for action, they would naturally wish to include in the training data the most recent available years rather than simply relying on model parameters that had been determined using old data. Taking also into account the arguments in Brandt (2011) that the choice of a size of training window can influence the final fit of a model, the estimation of reasonable values for *n*, the training window size, formed part of the project. A consequence of following this process is that we do not report specific model coefficients, because these are dependent on the reference year for which predictions are created.

**Algorithm 1 Identify predictors by AUPRC**

initialise *bestmeanAUPRC* to 0

initialise *candidates* to all possible candidate predictors

initialise *predictors* to ∅ {set of all predictors to be used by the model}

**repeat**

 set *prevmeanAUPRC* to *bestmeanAUPRC*

 set *candidatemeanAUPRCs* to ∅ {iterate for each candidate predictor, *cP*}

 **for** each *cP* in *candidates* **do**

  **for** *i* from 1 to *K* **do**

   randomly select, without replacement, 10 *referenceYears* from 1975 to 1999 inclusive

   **for** each *referenceYear* **do**

    train logistic model using 25 years data prior to *referenceYear* to predict onset of instabilities one year (one-year model) or two years (two-year model) after *referneceYear*

   **end for**

   calculate *iterAUPRC* over all predictions

  **end for**

  average over the K simulations to calculate *meanAUPRC*

  add (*cP*, *meanAUPRC*) to *candidateAUPRCs*

 **end for**

 add *cP* with highest *meanAUPRC* to *predictors*

 set *bestmeanAUPRC* equal to *meanAUPRC* for this predictor

**until** *t*.*test*(*bestmeanAUPRC* > *prevmeanAUPRC*) == *FALSE*

*candidates*—all candidate predictors

*predictors*—those predictors selected by the algorithm

*referenceYear*—the year in which a prediction is assumed to be made.

*iterAUPRC*—AUPRC for one iteration, one new candidate predictor.

*meanAUPRC*—mean of *K* *iterAUPRC* values, one new candidate predictor

*candidatemeanAUPRCs*—set of (*candidate*, *meanAUPRC*) pairs among which the best will be selected

*bestmeanAUPRC*—highest *meanAUPRC* of *candidatemeanAUPRCs* for the current round of predictor choice *prevmeanAUPRC*—*bestmeanAUPRC* for the previous round of predictor choice

### Model construction

We used logistic regression to construct two different models; these estimated the probability of an instability within one year and two years respectively after a reference year. Here and in the following sections the reference year is the year in which the prediction is hypothetically generated, based on data available up to but not including the reference year. We adopted this procedure because this is how the models would be used by an actual forecaster because, in practice, forecasters are forced to use slightly out-of-date information. In any given year, full-year data for a predictor can only be available up to the prior year, and any prediction of instability must be made, not for the current year (because that would not be a prediction) but for the following year or for the following two years for the one-year and two-year models respectively. Country-years with ongoing conflict were excluded from our predictions.

**Fig 1:**
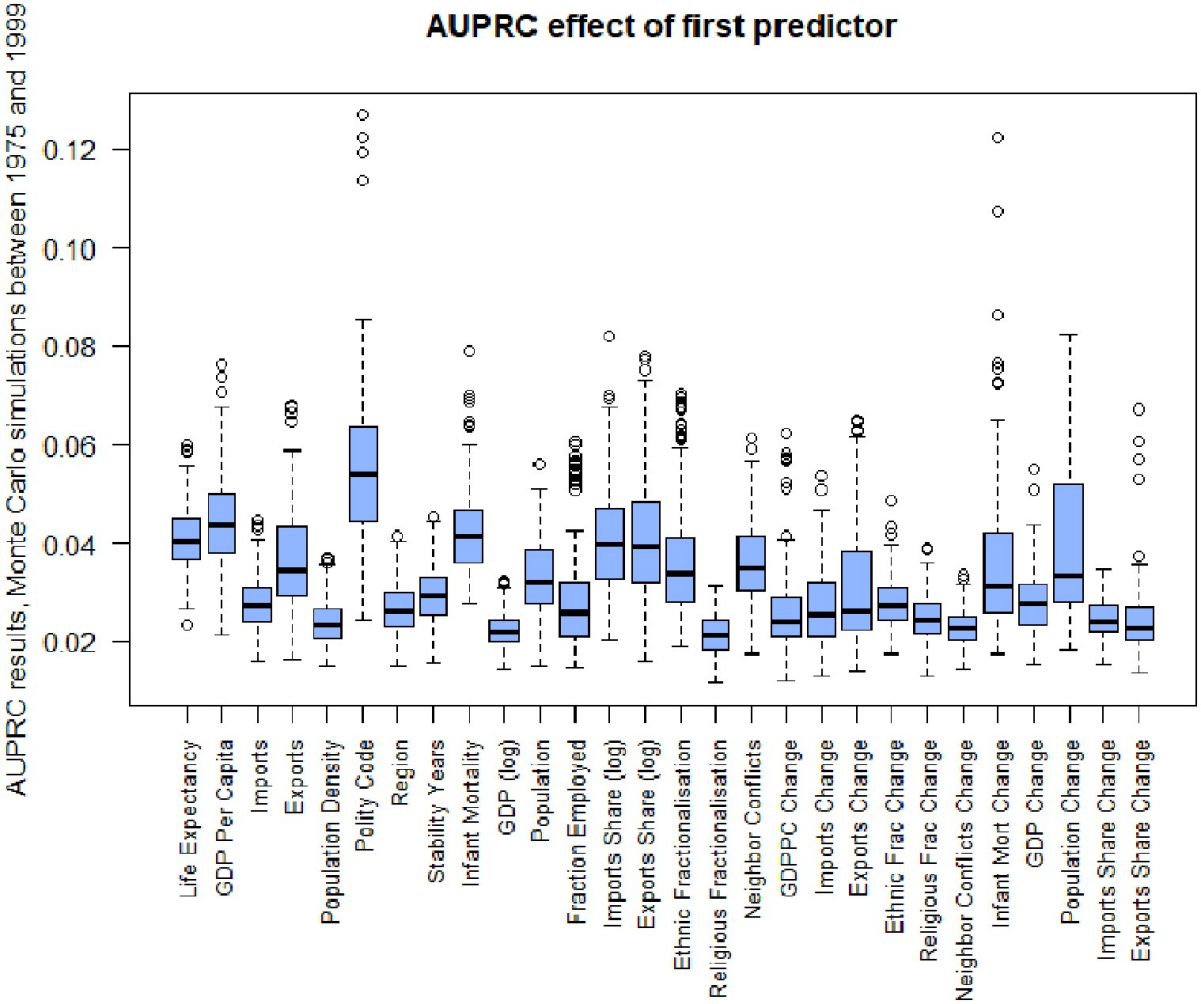
AUPRCs of single-variable models with a one year event horizon. Models were produced on reference years selected from 1975–1999. 95% CIs were calculated via Monte Carlo simulations. Circles indicate outliers.

We constructed these two models by incrementally adding predictors until we found that adding further predictors did not improve their predictive performance in a manner similar to that advocated by Rudin [5], as detailed in Algorithm 1. To start, we tested each potential predictor in a single-predictor model, using K rounds of Monte-Carlo simulation. In each round, we randomly selected 10 reference years from the period 1975—1999 to generate an AUPRC for each predictor. We repeated this process 200 times and used the resultant AUPRCs to identify the predictors that gave the highest mean AUPRC (Fig 1). This selection method is similar to the ‘bootstrap’ statistical estimation technique, but utilises each full data year rather than each individual country-year as the individually sampled unit. We chose the number of simulation rounds (K) to be 200 because increasing K further produced no difference in the estimates. Missing values were imputed using the imputeTS package in R using Kalman smoothing where possible, and interpolation otherwise [30]. Computing resources used were a standard desktop PC. Both the analysis code and the data can be found in an Open Science Framework (OSF) repository osf.io/3gr72.

*GDP per capita*, *life expectancy*, *infant mortality* and *polity code* have the highest individual predictive power. *Polity code* divides governments into five types—autocracy, partial autocracy, democracy, partial democracy and factionalised democracy [15]). These are based on data from the Polity IV dataset of the PITF, which was formerly known as the SFTF, and are a combination of measures of executive recruitment (democratic or otherwise) and competitiveness of elections (from suppressed to fully competitive). Factionalisation describes a state where competing blocks are sharply polarised and may involve intimidation and mass protests.

Subsequent iterations of the simulation process added one predictor to the model each round until the improvement in AUPRC was no longer statistically significant. The empirical mean, *μ*, and standard deviation, *σ*, of test AUPRCs generated by each successive model in 200 simulation runs are as shown in Table 2. For second and subsequent added predictors, a two-sample t-test was performed to determine if any improvement in test AUPRC from adding another predictor was statistically significant. Using this procedure we found that, for each model, AUPRC stopped improving after three predictors. For both of these models, the order of predictor was *polity code*, *infant mortality* and *years of stability*.

**Table 2:** Comparison of AUPRCs. *μ* and *σ* of AUPRCs generated from models trained on 200 simulated data sets for one- and two-year prediction periods. Each test set comprises a random selection of ten reference years drawn from the period 1975—1999 inclusive.

One-year model AUPRCs			Two-year model AUPRCs		
predictor	*μ*(*σ*)	p < 0.001	predictor	*μ*(*σ*)	p < 0.001
PolCode	0.055 (0.016)	Yes	PolCode	0.060 (0.018)	Yes
+ InfantMort	0.093 (0.032)	Yes	+ InfantMort	0.095 (0.034)	Yes
+ StabilityYears	0.108 (0.037)	Yes	+ StabilityYears	0.115 (0.041)	Yes
+ Δ Population	0.120 (0.038)	No	+ Δ Population	0.116 (0.038)	No
+ Δ InfantMort	0.116 (0.040)	No	+ Δ InfantMort	0.118 (0.042)	No

Why did the models use these particular predictors? As noted above, *GDP per capita*, *life expectancy*, *infant mortality* and *polity code* have among the highest individual predictive power. However, *GDP per capita*, *life expectancy* and *infant mortality* are each moderately or strongly correlated with each other, as shown by Table 3. Once one is chosen as a predictor, the others do not add much incremental predictive ability. Consequently, the models used only one of these predictors—in this case, *infant mortality*. Table 4 shows the point biserial correlations between levels of *polity code* and the other predictors used to construct our two models. As discussed later, we found that most instability events were associated with a polity code of factional democracy. As Table 4 shows, factional democracy had at most weak correlations with *infant mortality* or *years of stability*, thereby justifying the inclusion of polity code in our models. Table B in S1 Text lists the correlations between all potential predictors and Table C in S1 Text lists the biserial correlations between the potential predictors and polity code.

**Table 3:** Correlations between *life expectancy*, *GDP per capita* and *infant mortality (log)*.

	GDP per capita	Infant Mortality (log)
Life Expectancy	(***)	(***)
	0.517	-**0.864**
GDP Per Capita		(***)
		-0.532

Correlations between the levels of polity code and the predictors in the models (*** = p < 0.001). Moderate correlations (magnitude between 0.5 and 0.8) underlined. Strong correlation (magnitude greater than 0.8) underlined and bolded.

**Table 4:** Point biserial correlations between *polity code* and other selected predictors.

	Autocracy	Partial Autocracy	Factional Democracy	Partial Democracy	Democracy
Infant Mortality (log)	(***)	(***)	(***)	(***)	(***)
	0.356	0.226	0.05	**0.84**	-0.566
Years of Stability	(***)	(***)	(***)	(***)	(***)
	-0.145	-0.124	-0.074	0.055	0.281

Correlations between the levels of polity code and the predictors in the models (*** = p < 0.001). Moderate correlations (magnitude between 0.5 and 0.8) underlined. Strong correlation (magnitude greater than 0.8) underlined and bolded. The correlation between *infant mortality* and *years of stability* is a moderate correlation of -0.519.

A check on the usefulness of our method of model construction can be obtained by comparing its results with that obtained by penalised logistic regression. As detailed in S1 Fig, we used lasso regression with the same initial set of predictors to create models to predict instabilities one year in advance. However, when these one-year models were tested on a new selection of reference years from 2000–2015, they produced a mean AUPRC of only 0.038 (0.084 for a two-year model). By comparison, the mean AUPRC for our one-year and two-year models for these reference years were 0.078 and 0.084, as discussed below. So the lasso procedure never outperformed the iterative procedure and in addition selected a larger number of predictors: on average, on each run, 11 predictors had non-zero coefficients. Furthermore, each run produced a different set of predictors. Combined, these two factors made it difficult to explain the predictions made by lasso regression. In the absence of any increase in predictive capability, there seems not to be an advantage in using lasso regression.

### Training period length

So far we have been using a 25-year period of training data in order to build models, but is this the right choice? Using all historical data runs the risk that, with societal change having taken place over the decades, the effects of specific predictors may have changed in strength. For instance, new factors may arise which influence the chance of instability, such as the penetration of social media and mobile phones, which has been hypothesised as an influential factor in the Arab Spring of the early 2000s [31], and these new factors may reduce the predictive power of the previous predictors. Considerations such as this argue for using only the most recent data. On the other hand, too little data will lead to inconsistent results and badly-trained models, which argues for using as much historical data as possible. Brandt et al. [27] observed that “ambiguous or ad hoc guidelines for the choice of training sets” (p. 39) have been an issue in Political Science modelling that can interfere with the accurate identification of superior models and identified this as a relative weakness in Political Science research as compared to meteorology or macroeconomic forecasting.

**Fig 2:**
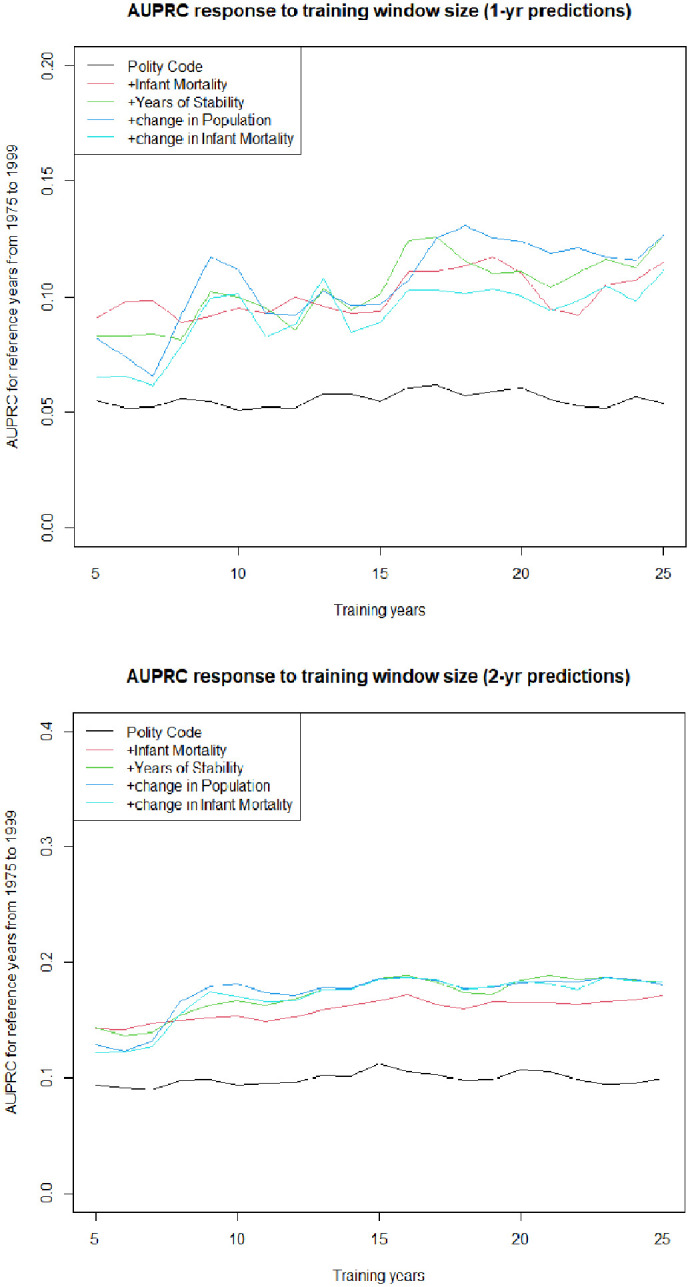
Response to training window size. Building up a 5-parameter model, one variable at a time, to predict instability one and two years in advance.

Fig 2 shows the effect of a range of training window sizes on the model’s predictive power, for reference years between 1975 and 1999, for both one-year and two-year predictions. In the case of one-year predictions, for the simplest model, there was little variation as function of training set size. With the addition of further parameters, best results are seen after about 16 years, with no obvious improvement beyond that point in using extra training data. The highest values for AUPRC for one-year predictions were seen between 17 and 25 years. This figure also shows the decline in predictive ability for the most complex of the models, confirming that for predicting instability one year in advance, a three-predictor model involving polity code, infant mortality and years of stability is the best choice. Given that there is no demonstrated advantage in using a longer training period, we concluded that a 17 year training period would be appropriate.

A three-predictor model can also be used to predict two years in advance, as shown in Table 2. This model has a slightly better predictive power and somewhat less variability than the one-year model. When we consider the best training interval for this data, in the same way that we have already done for one-year predictions, there is a maximum at 16 years for all model variants with more than one parameter, so this was the training period we subsequently used for the two-year model.

### Testing the model

In the initial model development, all model validation was performed on out-of-sample data in individual Monte Carlo simulation runs, using pre-2000 reference years. However, this means each pre-2000 country-year may at some point have been used for both fitting and testing. In order to perform a more rigorous test of the models, we now tested them using post-2000 reference years, as these reference years had not previously been used. We proceeded through the same steps as in the initial train/test process, predicting instability probabilities for each year in turn on the basis of the previous *n* years of data (with *n* now being set to 17 for the one-year model and 16 for the two-year model) and calculating a single AUPRC on the basis of this new set of predictions.

As shown in Table 2, for the reference years 1975–1999 inclusive, the AUPRC for the one-year model (*polity code* + *infant mortality* + *years of stability*) was 0.11 with 95% CI [0.04,0.18]. For the new reference years 2000–2015, the AUPRC for this model was 0.078. For the reference years 1975 to 1999 inclusive, the AUPRC for the two-year model was 0.12 with 95% CI [0.03,0.20]). For the new reference years 2000–2015, the AUPRC for this model was 0.084. For both the one-year and two-year model, the AUPRC value for the reference years 2000–2015 was within the predicted 95% CIs for the AUPRC value calculated for the reference period 1975–1999. This gives us confidence that our models are not overfitted, so their predictions will continue to be reliable for future years. It also indicates that the calculated CIs are reasonable. Finally, we note that the actual AUPRCs exhibit the same characteristic we found in the pre-2000 simulations: the two-year model has a slightly higher average AUPRC than the one-year model.

**Fig 3:**
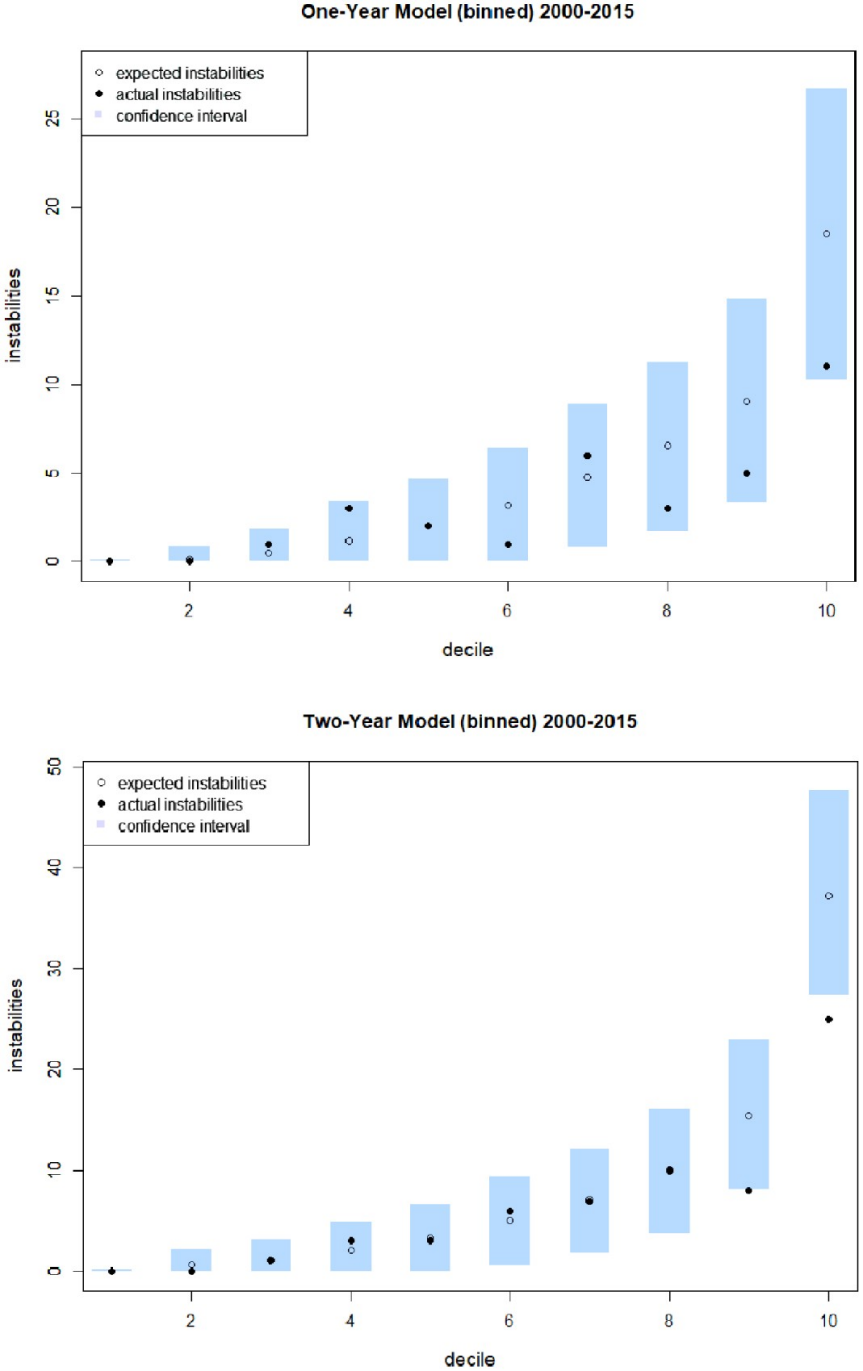
Binned expectation plots. Actual instability events (solid dots) and expectations (hollow dots) with 95% confidence interval at each point, grouped by deciles.

AUPRC alone will not tell us if the predicted probabilities in this model are in themselves accurate. Two models that rank the probabilities of events in the same order will always produce the same AUPRC value, even if the actual probabilities in one (or both) are wildly inaccurate [32]. The assessment of whether the predicted probabilities are accurate is best done by binning the data to show how the predicted number of instabilities compares to the actual instabilities. Binned plots for the one- and two-year models are shown in Fig 3. To produce these plots, country-years were ordered according to instability probability, and grouped by decile. For each decile, we calculated the expectation of the number of instabilities that the model predicts should have occurred among those country-years, together with a 95% confidence interval for the expectation. Most of the data is within these confidence intervals but there seems to be a tendency for both models to over-predict instability events in the higher deciles. This may be related to the fact that, in general, political instability was at its highest in the late 1960s and appears to be still declining from there.

### Explaining high-probability cases

A model’s prediction can best be explained by identifying which predictors were the most influential. The more a predictor increases the predicted probability of an instability, the more influential the predictor [33]. To illustrate this, we take as an example the predictions of the two-year model for the Central African Republic (CAR) in 2002 and Cote d’Ivoire in 2000, both of which entered political instability within two years of the prediction being made. Table 5 shows the predicted probability of instability according to the full three-factor model, as well as for all possible submodels. The table shows that the predicted probability of CAR’s instability falls significantly when removing *polity code*, to the point where a one-factor model with only *polity code* predicts a greater probability of instability than the two-factor model without it. We can therefore conclude that the primary reason why the two-year model predicted such a high probability of instability for CAR in 2002 was because of its *polity code* which, in 2002, was factional democracy. For Cote d’Ivoire, the explanation is somewhat different. Cote d’Ivoire, as an autocracy, actually receives almost the same instability probability if we leave aside its *polity code*, because partial autocracies are not particularly prone to enter instability. Thus, *polity code* is not the reason why Cote d’Ivoire was given a high chance of entering instability. The high predicted probability was caused by a combination of the other two factors, *infant mortality* and *years of stability*. Out of these two factors we see that *infant mortality* was the most important. A one factor model that used just *infant mortality* predicted almost the same probability of instability as the full three factor model. This shows that the primary reason for the predicted probability of instability was the high infant mortality rate.

**Table 5:** Example predicted instability probabilities. Predicted probability of instability within two years for CAR and Cote d’Ivoire made by the full three predictor model and a series of nested models created by removing either one, two or three predictors from the full model (with 95% CI).

Model used	CAR 2002	Cote d’Ivoire 2000
Full model	**0.30, [0.20,0.43]**	**0.10, [0.07,0.15]**
Polity Code + Infant Mortality only	0.23, [0.17,0.31]	0.08, [0.06,0.11]
Polity Code + Stability Years only	0.17, [0.11,0.25]	0.07, [0.05,0.10]
Infant Mortality + Stability Years only	0.11, [0.08,0.15]	0.11, [0.08,0.15]
Polity Code only	0.15, [0.11,0.20]	0.06, [0.04,0.08]
Infant Mortality only	0.09, [0.08,0.12]	0.09, [0.07,0.12]
Stability Years	0.05, [0.03,0.06]	0.04, [0.03,0.06]
Intercept only	0.05, [0.04,0.06]	0.05, [0.04,0.06]

To drill down further, we can consider counterfactual polity codes. Table 6 shows what the instability probabilities would have been under the four other polity codes, according to the full three-factor two-year model. This table shows that had CAR been a democracy, then the predicted probability of instability within the next two years would have been just 3%, as opposed to the actual prediction of 30%. This table shows that, out of all the polity codes, CAR’s *polity code* of factional democracy corresponded to the highest predicted instability. This is further evidence that the primary reason what the two-year model predicted such a high probability of instability for CAR in 2002 was because of its *polity code*. The specific values for the probability of instability under various polity codes are consistent with existing research showing that while democracies are generally less likely to transition into instability than non-democracies, states in transition to full democracy (i.e. factional democracies) are generally more likely to do so [34].

**Table 6:** Counterfactuals. Actual prediction (bold) versus counterfactual predictions for different polity codes with 95% CI for CAR and Cote d’Ivoire, two-year event horizon.

Polity code	CAR 2002	Cote d’Ivoire 2000
Factional Democracy	**0.30, [0.20,0.43]**	0.31, [0.20,0.43]
Democracy	0.03, [0.01,0.14]	0.02, [0.01,0.11]
Partial Democracy	0.03, [0.01,0.08]	0.05, [0.02,0.10]
Partial Autocracy	0.09, [0.06,0.15]	0.10, [0.06,0.16]
Autocracy	0.12, [0.06,0.15]	**0.10, [0.07, 0.15]**

Turning our attention to Cote d’Ivoire, the numbers tell a different story. As an autocracy, Cote d’Ivoire had a predicted probability of instability of 10%. Had it been a factional democracy this would have risen to 31%. Conversely, had it been a democracy this would have dropped to just 2%. Thus, while *polity code* had the potential to significantly affect the predicted probability of instability, in this case it did not. As discussed above, for Cote d’Ivoire in 2000, the most significant predictor was *infant mortality*. This shows that *polity code* is not always a significant predictor.

We should also consider whether we are justified in creating these counterfactuals. Given that our model is trained over a specific set of data (in particular, one in which there is a moderately strong correlation between democracy and life expectancy), should we expect it to perform well when assessing counterfactuals based on changes in *polity code*? To answer this question, King and Zeng [35] assert that we need to check the convex hull of the data on which the model was trained (the smallest possible area which is both convex and contains all the data) to determine if a counterfactual prediction can justifiably be made. If the counterfactual is within the convex hull of the data, the counterfactual is based on interpolation rather than extrapolation, and is more likely to be accurate. The principle here is to try to avoid making strong claims about our predictions in regions of the data where we do not have any explicit information—for instance, we can make good predictions about high income autocracies because there are many of them in the Gulf States to train our model with, and good predictions about high income democracies because there are many of them worldwide—but not about high income factional democracies because there are none in our data. In the specific example of CAR and Cote d’Ivoire, the counterfactuals of changing *polity code* are within the convex hull of the data except in the case of democracy or partial autocracy. Therefore we can have high confidence in most of the potential counterfactuals but there is a larger degree of uncertainty regarding the specific predictions for democracy. The ability to meet this criterion when generating counterfactuals for explanation is another advantage of a simpler model, because a greater proportion of possible counterfactuals will be within the convex hull of the data when the dimension of the space is small. In the case of the data for Cote d’Ivoire, counterfactual analysis tells us that it would be advantageous to encourage a partial degree of democracy. Unsurprisingly, the model predicts that a factional democracy here would be extremely dangerous.

## Discussion

In this investigation we created models to predict instabilities one and two years in advance. We found that both models used just three predictors: *polity code*, *infant mortality* and *years of stability*. The fact that both models found the same predictors gives us confidence in these predictors.

For most of our predictions, we found that the most important predictor in our model was *polity code*. Consistent with other research [36, 37], we find that instability is most likely for country-years with a polity code of factional democracy which is our shorthand for what Goldstone et al. [15] describe as ‘partial democracy with factionalism’. This is a dummy variable that Goldstone et al. derived from two POLITY IV scales, the Executive Recruitment (EXREC) and the Competitiveness of Political Participation (PARCOMP) scales. Partial democracy with factionalism is defined as a simultaneous rating of 6–8 on the EXREC scale, which corresponds to the categories of ‘Ascription and Election’, ‘Transitional or Restricted Election’ and ‘Competitive Election’ and a rating of ‘Factional’ on the PARCOMP scale. For this scale, ‘Factional’ is defined as ‘Polities with parochial or ethnic-based political factions that regularly compete for political influence in order to promote particular agendas and favor group members to the detriment of common, secular, or cross-cutting agendas’ [38].

It has been suggested that because this definition of factionalism includes low-level violence, such as voter intimidation, it should not be used to predict political instability because this usually involves some form of violence [39]. It has been claimed that doing so effectively amounts to predicting violence on the basis of already existing violence. Goldstone et al. [15] addressed this issue by measuring the degree to which the predictive power of polity code would be reduced by incorporating explicit measures of civil violence and civil protest into their model. Since no significant reduction in the predictive power of polity code was observed, they concluded that polity code was not acting as a proxy for violence or political unrest, thereby justifying the inclusion of polity code in their model. A second potential issue with the use of the PITF data is that the Adverse Regime Change event is somewhat biased by Polity Code, since a transition in favour of democracy is never coded as ‘Adverse’. However, as long as all polity variables are independently determined before a prediction is made, this will not affect our evaluation of the degree of predictive success of the model, though it may affect our evaluation of which predictors could be considered the root causes of this instability.

Our models also predict that the probability of instability increases with increasing *infant mortality* and *years of stability*. In neither case are we claiming that there is necessarily a causal relationship. For example, as shown in Table B in S1 Text, *infant mortality* is correlated with a number of factors, most notably *life expectancy* and *GDP per capita*. As such, it is probably a proxy measure for something akin to *existential security*, the degree to which basic human needs are satisfied [40]. Similarly, at first glance, it may be surprising that both models predict that the probability of instability *increases* with increasing *years of stability*, though this finding is broadly in agreement with previous work [14]. A possible reason for this is that it is more likely that a country that has recently left a period of instability is less able to enter into another instability due to a depletion of the human capital and other resources it would need to do so.

In this research, we have confined our attention solely to macroeconomic factors. While we have demonstrated that we can make considerable progress using only these factors, there are a range of additional predictors that could also be used such as fine-grained geographic data [12] and natural language communications, particularly those of political leaders [13]. Incorporating these additional predictors may allow us to increase our predictive power. Further research would be needed to determine the best way to do this.

Finally, we should consider how our model should be used by policy makers. Because our model focuses as much on the transparency of its predictions as on the accuracy of its predictions, its AUPRC is not as high as the state-of-the-art ensemble models that have emphasized only accuracy (e.g. [3]). We note that since the AUPRC of our models is still high relative to these more advanced models, for many country-year instances the predicted probabilities of instability for our models would be similar those made by the more advanced models. Thus, our models can be used to explain the predictions of these more advanced models. The advantage of this approach is that the predictions of our models can be fully explained in a simple and transparent manner, whereas the predictions of these more advanced models are less transparent [4].

In conclusion, we have shown how models that predict political instability with one-year and two-year event horizons can be constructed in a way that allows their predictions to be easily explained. Using these models, we can predict which countries at which times are likely to experience political instability and we can further explain why instability is predicted for a particular country-year combination. While there are a number of outstanding research issues that still need to be addressed, even in their current form these models are practically useful in that they can be combined with the state-of-the-art models to provide a degree of transparency that is currently lacking in the field [4, 5].

## Supporting information

S1 Text Candidate predictors and correlations. (PDF)

S1 Fig Lasso regression comparison. (PDF)
