# Supplementary material for: Explainable models for forecasting the emergence of political instability
Source: PLoS One. 2021 Jul 29;16(7):e0254350. doi: 10.1371/journal.pone.0254350 (PMC8321219; doi:10.1371/journal.pone.0254350)
Supplement: S1 Text — (PDF) [file pone.0254350.s001.pdf]

**Table A. Candidate predictors.**

| ID | Predictor | Source | Notes |
|---|---|---|---|
| 1 | Life expectancy | World Bank[a] | |
| 2 | GDP per capita (PPP) | World Bank[a] | PPP (constant 2011 $) |
| 3 | Imports | World Bank[a] | Imports of goods and services (% of GDP) |
| 4 | Exports | World Bank[a] | Exports of goods and services (% of GDP) |
| 5 | Population density | World Bank[a], UN[b] | |
| 6 | Polity code | Center for Systemic Peace [1] | |
| 7 | Region | Replication Data [2] | |
| 8 | Years of stability | PITF Conflict Data | |
| 9 | Infant mortality (log) | Abouharb [3], World Bank[a] | |
| 10 | Total GDP (log) | Penn World Tables[c] | Expenditure-side real GDP at chained PPPs (in million 2011 USD) |
| 11 | Population (log) | Penn World Tables[c], World Bank[a], UN[b] | |
| 12 | Fraction Employed | Penn World Tables[c] | |
| 13 | GDP share of imports (log) | Penn World Tables[c] | Share of merchandise imports at current PPPs |
| 14 | GDP share of exports (log) | Penn World Tables[c] | Share of merchandise exports at current PPPs |
| 15 | Degree of ethnic fractionalisation | Cline Center For Advanced Social Research [4] | |
| 16 | Degree of religious fractionalisation | Cline Center For Advanced Social Research [4] | |
| 17 | Conflicts in neighboring countries | Correlates of War project [5] | |
| 18 | Change in GDP per capita (PPP) | | proportional difference from previous year |
| 19 | Change in imports | | proportional difference from previous year |
| 20 | Change in exports | | proportional difference from previous year |
| 21 | Change in ethnic fractionalisation | | proportional difference from previous year |
| 22 | Change in religious fractionalisation | | proportional difference from previous year |
| 23 | Change in neighbor conflicts | | absolute difference from previous year |
| 24 | Change in infant mortality | | absolute difference from previous year |
| 25 | Change in GDP | | absolute difference from previous year |
| 26 | Change in population | | absolute difference from previous year |
| 27 | Change in share of exports | | absolute difference from previous year |
| 28 | Change in share of imports | | absolute difference from previous year |

[a]Available at https://data.worldbank.org/
[b]Available at http://data.un.org
[c]Version 9.1 - available at https://www.rug.nl/ggdc/productivity/pwt or in R package pwt9

## Table B. Correlations between all candidate predictors.

| | Life Expectancy | GDP per capita | Infant Mortality (log) | Imports | Exports | Imports share (log) | Exports Share (log) | Total GDP (log) | Population (log) | Population Density | Stability Years | Ethnic Frac | Religious Frac | Neighbor Conflicts | GDP per capita increase | Infant Mortality increase | Imports increase | Exports increase | Imports share increase | Exports Share increase | Total GDP increase | Population increase | Ethnic Frac increase | Religious Frac increase | Neighbor Conflicts increase |
|---|---|---|---|---|---|---|---|---|---|---|---|---|---|---|---|---|---|---|---|---|---|---|---|---|---|
| Life Expectancy | (***) 1 | (***) 0.517 | (***) **-0.864** | (***) 0.154 | (***) 0.268 | (***) 0.339 | (***) 0.348 | (***) 0.574 | (***) 0.159 | (***) 0.167 | (***) 0.409 | (***) -0.374 | (***) -0.037 | (***) -0.152 | -0.006 | (***) -0.074 | -0.02 | (*) -0.028 | 0.005 | 0.003 | (***) 0.041 | (***) -0.309 | (***) -0.109 | (***) -0.087 | -0.021 |
| GDP per capita | | (***) 1 | (***) -0.532 | (***) 0.09 | (***) 0.361 | (***) 0.429 | (***) 0.299 | (***) 0.415 | (***) -0.066 | (***) 0.182 | (***) 0.321 | (***) -0.152 | (*) -0.023 | (***) -0.202 | 0.014 | (***) -0.041 | -0.013 | -0.016 | -0.012 | -0.017 | (**) 0.029 | (***) 0.087 | (***) -0.142 | (***) -0.149 | -0.007 |
| Infant Mortality (log) | | | (***) 1 | (***) -0.167 | (***) -0.309 | (***) -0.438 | (***) -0.434 | (***) -0.58 | (***) -0.148 | (***) -0.218 | (***) -0.519 | (***) 0.31 | (***) -0.086 | (***) 0.205 | 0.005 | (***) 0.124 | (*) 0.026 | (**) 0.031 | -0.005 | 0.001 | -0.021 | (***) 0.371 | (***) 0.117 | (***) 0.096 | 0.02 |
| Imports | | | | (***) 1 | (***) 0.794 | (***) 0.297 | (***) 0.42 | (***) -0.271 | (***) -0.438 | (***) 0.386 | (***) 0.156 | (***) 0.055 | (***) 0.137 | (***) -0.046 | (*) -0.024 | -0.014 | (***) -0.071 | -0.013 | -0.01 | (*) 0.024 | 0.018 | (**) 0.031 | (***) 0.105 | (***) 0.167 | 0.005 |
| Exports | | | | | (***) 1 | (***) 0.512 | (***) 0.45 | (***) -0.061 | (***) -0.368 | (***) 0.464 | (***) 0.261 | (***) 0.056 | (***) 0.126 | (***) -0.08 | (***) -0.045 | -0.019 | (**) -0.036 | (***) -0.071 | 0.008 | 0.004 | (***) 0.075 | (***) 0.059 | (***) 0.069 | (***) 0.126 | -0.002 |
| Imports share (log) | | | | | | (***) 1 | (***) **0.817** | (***) 0.176 | (***) -0.164 | (***) 0.191 | (***) 0.315 | (*) -0.025 | (***) 0.099 | (***) -0.143 | 0.000 | (*) -0.027 | -0.003 | (**) -0.033 | (***) 0.081 | (*) 0.026 | (***) 0.075 | (***) -0.059 | (***) -0.041 | (**) -0.033 | -0.009 |
| Exports Share (log) | | | | | | | (***) 1 | (**) 0.035 | (***) -0.251 | (***) 0.211 | (***) 0.269 | (***) -0.065 | (***) 0.102 | (***) -0.165 | -0.002 | (**) -0.035 | -0.021 | -0.008 | 0.015 | (***) 0.057 | (***) 0.073 | (***) -0.103 | (***) -0.077 | 0.006 | -0.011 |
| Total GDP (log) | | | | | | | | (***) 1 | (***) 0.756 | (***) 0.084 | (***) 0.235 | (***) -0.228 | (**) -0.031 | 0.02 | 0.014 | (***) -0.039 | (*) -0.028 | (*) -0.024 | 0.007 | 0.002 | (**) 0.03 | (***) -0.242 | (***) -0.162 | (***) -0.045 | -0.007 |
| Population (log) | | | | | | | | | (***) 1 | 0.002 | (***) -0.053 | (***) -0.098 | -0.013 | (***) 0.199 | 0.019 | -0.007 | (*) -0.024 | -0.008 | 0.017 | 0.016 | 0.006 | (***) -0.188 | (***) -0.129 | (***) 0.037 | -0.004 |
| Population Density | | | | | | | | | | (***) 1 | (***) 0.041 | (***) -0.071 | (***) 0.188 | (***) -0.083 | -0.004 | -0.014 | 0.00 | -0.002 | -0.005 | -0.011 | (***) 0.045 | -0.01 | (*) -0.024 | 0.022 | -0.006 |
| Stability Years | | | | | | | | | | | (***) 1 | (*) -0.022 | (***) 0.102 | (***) -0.108 | 0.003 | (*) -0.027 | 0.004 | -0.002 | -0.004 | -0.003 | -0.009 | (***) -0.104 | (**) -0.034 | (***) -0.057 | -0.012 |
| Ethnic Frac | | | | | | | | | | | | (***) 1 | (***) 0.26 | (***) 0.163 | 0.003 | (*) 0.026 | 0.02 | (*) 0.026 | -0.013 | 0.002 | 0.00 | (***) 0.253 | (***) 0.352 | (***) 0.097 | 0.001 |
| Religious Frac | | | | | | | | | | | | | (***) 1 | (***) -0.054 | -0.009 | 0.004 | 0.016 | 0.018 | 0.001 | -0.003 | 0.004 | -0.007 | (***) -0.058 | (***) 0.124 | -0.008 |
| Neighbor Conflicts | | | | | | | | | | | | | | (***) 1 | 0.007 | 0.003 | 0.014 | 0.015 | -0.006 | -0.004 | 0.017 | (***) 0.166 | (***) 0.118 | (***) 0.149 | (***) 0.218 |
| GDP per capita increase | | | | | | | | | | | | | | | (***) 1 | -0.019 | -0.002 | 0.009 | -0.011 | -0.008 | (***) -0.066 | (*) -0.024 | -0.007 | -0.006 | 0.002 |
| Infant Mortality increase | | | | | | | | | | | | | | | | (***) 1 | 0.005 | -0.003 | 0.00 | 0.002 | (**) -0.034 | 0.008 | 0.009 | 0.009 | -0.003 |
| Imports increase | | | | | | | | | | | | | | | | | (***) 1 | (***) 0.451 | (*) -0.028 | (***) -0.209 | (***) 0.101 | -0.011 | 0.019 | 0.004 | -0.019 |
| Exports increase | | | | | | | | | | | | | | | | | | (***) 1 | (***) -0.189 | -0.001 | (***) -0.106 | 0.008 | (**) 0.029 | 0.014 | -0.01 |
| Imports share increase | | | | | | | | | | | | | | | | | | | (***) 1 | (***) 0.543 | -0.014 | -0.016 | 0.003 | -0.002 | -0.01 |
| Exports Share increase | | | | | | | | | | | | | | | | | | | | (***) 1 | (***) -0.09 | -0.018 | 0.019 | 0.002 | -0.012 |
| Total GDP increase | | | | | | | | | | | | | | | | | | | | | (***) 1 | (***) 0.103 | -0.007 | 0.005 | -0.009 |
| Population increase | | | | | | | | | | | | | | | | | | | | | | (***) 1 | (***) 0.055 | (***) 0.042 | 0.013 |
| Ethnic Frac increase | | | | | | | | | | | | | | | | | | | | | | | (***) 1 | (***) 0.114 | 0.002 |
| Religious Frac increase | | | | | | | | | | | | | | | | | | | | | | | | (***) 1 | 0.009 |

Correlations between predictors, with associated significance indicators (* : p < 0.05, ** : p < 0.01, *** : p < 0.001. Strong correlations (magnitude over 0.8) [6] bolded and underlined, moderate correlations (magnitude between 0.5 and 0.8) underlined only. Predictors chosen for the final models are shown in grey.

**Table C. Point biserial correlations between the levels of *Polity Code* and other candidate predictors.**

| | Autocracy | Partial Autocracy | Factional Democracy | Partial Democracy | Democracy |
|---|---|---|---|---|---|
| Life Expectancy | (***) -0.307 | (***) -0.234 | -0.009 | (***) 0.072 | (***) 0.5 |
| GDP per capita | (***) -0.047 | (***) -0.19 | (***) -0.143 | (***) -0.117 | (***) 0.43 |
| Infant Mortality (log) | (***) 0.356 | (***) 0.226 | (***) 0.05 | (***) -0.084 | (***) -0.566 |
| Imports | (***) -0.063 | -0.015 | (***) 0.078 | (***) 0.072 | (**) -0.034 |
| Exports | (***) -0.078 | (*) -0.026 | 0.017 | (**) 0.032 | (***) 0.07 |
| Imports share (log) | (***) -0.225 | (***) -0.042 | (**) -0.034 | (**) 0.036 | (***) 0.281 |
| Exports Share (log) | (***) -0.283 | (***) -0.06 | 0.004 | (***) 0.103 | (***) 0.276 |
| Total GDP (log) | (***) -0.249 | (***) -0.124 | (**) -0.03 | (**) 0.032 | (***) 0.384 |
| Population (log) | (***) -0.087 | (**) -0.031 | 0.015 | (***) 0.042 | (***) 0.078 |
| Population Density | (***) -0.077 | (***) -0.06 | -0.005 | (***) 0.162 | 0.004 |
| Stability Years | (***) -0.145 | (***) -0.124 | (***) -0.074 | (***) 0.055 | (***) 0.281 |
| Ethnic Frac | (***) 0.058 | (***) 0.116 | (***) 0.088 | (***) 0.071 | (***) -0.296 |
| Religious Frac | -0.013 | (***) -0.037 | 0.002 | (***) 0.073 | -0.016 |
| Neighbor Conflicts | (***) 0.082 | (***) 0.135 | (***) 0.041 | 0.001 | (***) -0.246 |
| GDP per capita increase | -0.002 | 0.004 | -0.001 | -0.008 | 0.006 |
| Infant Mortality increase | (*) 0.028 | 0.012 | -0.004 | -0.015 | (*) -0.026 |
| Imports increase | 0.016 | 0.007 | -0.001 | 0.003 | (*) -0.026 |
| Exports increase | 0.021 | 0.005 | -0.003 | 0.008 | (**) -0.033 |
| Imports share increase | 0.014 | -0.004 | (*) -0.026 | 0.008 | -0.001 |
| Exports Share increase | 0 | 0.005 | -0.005 | 0.013 | -0.012 |
| Total GDP increase | (**) -0.032 | (*) 0.024 | -0.009 | (***) 0.05 | (*) -0.024 |
| Population increase | (***) 0.219 | (***) 0.136 | -0.014 | (***) -0.062 | (***) -0.304 |
| Ethnic Frac increase | (***) 0.049 | (**) 0.031 | (***) 0.076 | (***) 0.05 | (***) -0.181 |
| Religious Frac increase | (***) 0.088 | (***) 0.067 | (*) 0.025 | (***) 0.051 | (***) -0.221 |
| Neighbor Conflicts increase | (*) 0.024 | 0.011 | -0.02 | -0.017 | -0.008 |

## References


1. Center for Systemic Peace. INSCR data page; 2020. http://www.systemicpeace.org/inscrdata.html.

2. Kennedy R. Making useful conflict predictions. Journal of Peace Research. 2015;52(5):649–664. doi:10.1177/0022343315585611.

3. Abouharb MR, Kimball AL. Special Data Feature A New Dataset on Infant Mortality Rates, 1816-2002. Journal of Peace Research. 2007;44(6):743–754. doi:10.1177/0022343307082071.

4. Cline Center For Advanced Social Research. Composition of Religious and Ethnic Groups (CREG) Project; 2020. https://clinecenter.illinois.edu/project/Religious-Ethnic-Identity/composition-religious-and-ethnic-groups-creg-project.

5. Correlates of War project. Direct Contiguity Data, 1816-2016. Version 3.2.; 2020. https://correlatesofwar.org/data-sets/direct-contiguity.

6. Samuel M, Okey LE. The Relevance and Significance of Correlation in Social Science Research. International Journal of Sociology and Anthropology Research. 2015;1(3):22–28.
