# Supplementary material for: Explainable models for forecasting the emergence of political instability
Source: PLoS One. 2021 Jul 29;16(7):e0254350. doi: 10.1371/journal.pone.0254350 (PMC8321219; doi:10.1371/journal.pone.0254350)
Supplement: S1 Fig — (PDF) [file pone.0254350.s002.pdf]

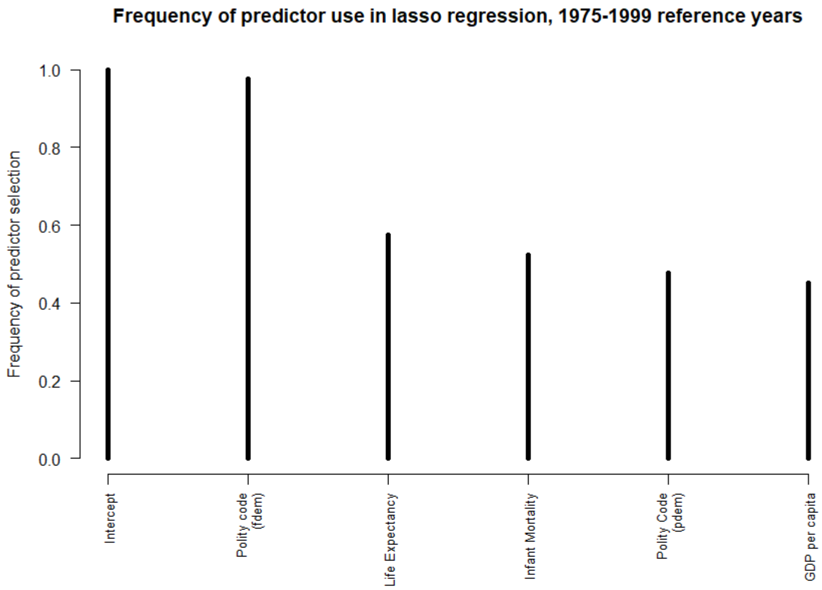

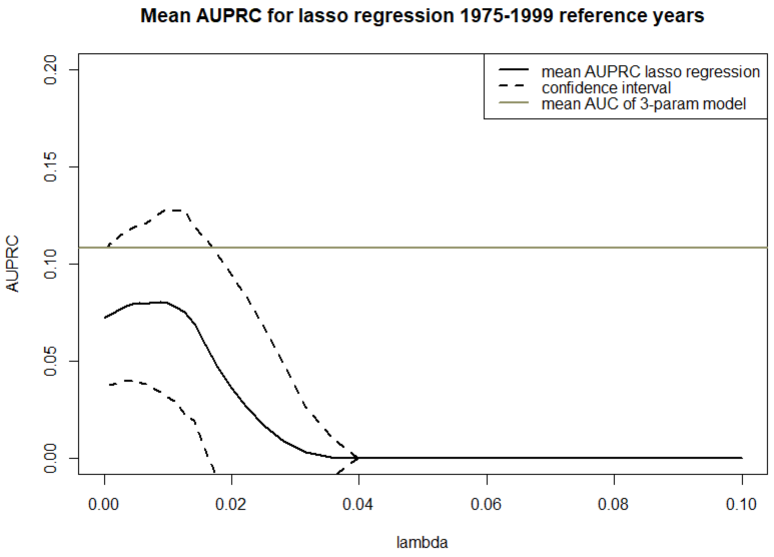

S1 Figure. Selected predictors and AUPRC results for lasso regression. This figure summarises the predictive ability of a one-year model selected by lasso regression, rather than by maximising AUPRC for successive individual predictors. Multiple versions of this model were trained using 30 years of data prior to the reference year using references years in the range 1975-1999 and tested on the year immediately after the reference year. We used a slightly longer training period than before because lasso regression benefits from using larger training sets because it considers all predictors simultaneously. Each new data subset produced a slightly different model, with non-zero coefficients for an average of 11 predictors. As Polity Code is categorical, each level was coded as a separate predictor. The most frequently used predictors include some overlap between predictors selected in 3-predictor AUPRC-selected models. However, they are not selected consistently for each new data subset, and the resulting models have reduced predictive performance compared to the 3-predictor model.
